# Non-viral nitric oxide-based gene therapy improves perfusion and liposomal doxorubicin sonopermeation in neuroblastoma models

**DOI:** 10.7150/thno.81700

**Published:** 2023-06-04

**Authors:** Aditi Bellary, Chance Nowak, Isabella Iwanicki, Fernando Flores-Guzman, Lydia Wu, Jessica J. Kandel, Theodore W. Laetsch, Leonidas Bleris, Sonia L. Hernandez, Shashank R. Sirsi

**Affiliations:** 1 Department of Biomedical Engineering, University of Texas at Dallas, Richardson, TX, USA.; 2 Department of Biological Sciences, University of Texas at Dallas, Richardson, TX, USA.; 3 Department of Surgery, University of Chicago Medical School, Chicago, IL, USA.; 4 Division of Oncology and Center for Childhood Cancer Research, Children's Hospital of Philadelphia and the Department of Pediatrics and Abramson Cancer Center, University of Pennsylvania, Philadelphia, PA, USA.

**Keywords:** gene therapy, inducible nitric oxide synthase (iNOS), sonopermeation, neuroblastoma, and quantitative contrast-enhanced ultrasound (qCEUS)

## Abstract

Neuroblastoma (NB) is a pediatric malignancy that accounts for 15% of cancer-related childhood mortality. High-risk NB requires an aggressive chemoradiotherapy regimen that causes significant off-target toxicity. Despite this invasive treatment, many patients either relapse or do not respond adequately. Recent studies suggest that improving tumor perfusion can enhance drug accumulation and distribution within the tumor tissue, potentially augmenting treatment effects without inflicting systemic toxicity. Accordingly, methods that transiently increase tumor perfusion prior to treatment may help combat this disease. Here, we show the use of gene therapy to confer inducible nitric oxide synthase (iNOS) expression solely in the tumor space, using focused ultrasound targeting. NOS catalyzes the reaction that generates nitric oxide (NO), a potent endogenous vasodilator. This study reports the development of a targeted non-viral image-guided platform to deliver iNOS-expressing plasmid DNA (pDNA) to vascular endothelial cells encasing tumor blood vessels. Following transfection, longitudinal quantitative contrast-enhanced ultrasound (qCEUS) imaging revealed an increase in tumor perfusion over 72 h, attributed to elevated intratumoral iNOS expression.

**Methods**: To construct a gene delivery vector, cationic ultrasound-responsive agents (known as “microbubbles”) were employed to carry pDNA in circulation and transfect tumor vascular endothelial cells *in vivo* using focused ultrasound (FUS) energy. This was followed by liposomal doxorubicin (L-DOX) treatment. The post-transfection tumor response was monitored longitudinally using qCEUS imaging to determine relative changes in blood volumes and perfusion rates. After therapy, *ex vivo* analysis of tumors was performed to examine the bioeffects associated with iNOS expression.

**Results**: By combining FUS therapy with cationic ultrasound contrast agents (UCAs), we achieved selective intratumoral transfection of pDNA encoding the iNOS enzyme. While transitory, the degree of expression was sufficient to induce significant increases in tumoral perfusion, to appreciably enhance the chemotherapeutic payload and to *extend survival time* in an orthotopic xenograft model.

**Conclusion**: We have demonstrated the ability of a novel targeted non-viral gene therapy strategy to enhance tumor perfusion and improve L-DOX delivery to NB xenografts. While our results demonstrate that transiently increasing tumor perfusion improves liposome-encapsulated chemotherapeutic uptake and distribution, we expect that our iNOS gene delivery paradigm can also significantly improve radio and immunotherapies by increasing the delivery of radiosensitizers and immunomodulators, potentially improving upon current NB treatment without concomitant adverse effects. Our findings further suggest that qCEUS imaging can effectively monitor changes in tumor perfusion *in vivo*, allowing the identification of an ideal time-point to administer therapy.

## Introduction

Neuroblastoma (NB) is the most common extracranial solid tumor afflicting infants and children 1. Current standard-of-care for high-risk disease includes surgical resection, intensive chemoradiotherapy, stem cell transplantation, and immunotherapy 2,3. Unfortunately, the estimated 5-year survival rate for patients with high-risk NB is only 50% 4, and those that achieve cure often manifest chronic adverse effects (including infertility, hearing loss, cardiovascular complications and poor growth) which emanate from off-target toxicities unleashed by the treatment itself 5,6. This grim reality highlights the need for targeted approaches to treat this malignancy, ideally increasing the anti-tumor efficacy while limiting systemic toxicity.

Poor prognoses in many cancers are often correlated with high levels of hypoxia and increased interstitial hypertension 7-9. Improving perfusion in tumors has the potential to reduce these incidences in solid tumors, leading to improved drug delivery and better therapeutic outcomes 10. Current approaches to restoring perfusion use anti-angiogenic agents (such as bevacizumab) to 'normalize' tumors by curbing unchecked vessel formation, thereby restoring the aberrant structure and function of the tumor vasculature 11,12. However, clinical trials have uncovered major challenges using anti-angiogenic strategies 13-15, most notably that acquired resistance and amplified invasiveness may ensue from blocking the VEGF pathway. Other approaches targeting the tumor vasculature to improve perfusion, such as Notch blockade, have led to accelerated metastasis 16. The prevailing explanation for this cascade of events posits that an incomplete response to anti-angiogenic therapy likely arises from limited bioavailability of therapeutic agent in the tumor mass 17, meaning that insufficient perfusion ultimately impedes intratumoral deposition.

Nitric oxide (NO) therapy has been proposed as a potential solution to overcome tumor hypoxia and poor perfusion 18,19. However NO-based strategies have met with only partial success for myriad reasons, and the effects of NO can be unpredictable. For instance, the literature extensively documents NO's paradoxical and context-dependent activity 20: at low concentrations NO boosts carcinogenesis, angiogenesis, and tumor proliferation, while at high concentrations it assists tumor regression by inducing extensive DNA damage, thus shunting cells toward apoptosis. Hence, delivering the precise concentration of NO to elicit a therapeutic response is critical. More importantly, NO is readily scavenged by hemoglobin in erythrocytes; in fact, it has a much greater affinity for hemoglobin than does oxygen and its half-life in whole blood is a mere 1.8 ms 21.

Given its short half-life and role as a ubiquitous signaling molecule, elevating systemic NO levels is neither practical nor desirable. Thus, strategies to exploit NO as a cancer therapy require localized delivery or production within the tumor space to restrict its bioeffects to this region. Several recent studies have developed NO carriers to improve the molecule's circulation half-life and efficacy, such as organic nitrates, nitrosometal complexes, N-diazeniumdiolates, furoxans, nitrosothiols, RRX-001 and L-arginine (see 22 for a comprehensive review). Polymeric nanoparticle-based NO donors have also been shown to enhance tumor perfusion and to increase permeability and retention in cancer therapy 23,24. However, the lack of targeting capabilities and propensity for off-target accumulation of these donors may curtail their viability *in vivo*.

To circumvent the abovementioned constraints, our approach delivers inducible nitric oxide synthase (iNOS) to tumor vascular endothelial cells non-virally using ultrasound guidance. Nitric oxide synthases are a class of enzymes that produce NO from oxidation of the substrate L-arginine in a nicotinamide adenine dinucleotide phosphate (NADPH)-dependent reaction 25. The iNOS variant of nitric oxide synthase is the most potent form and is not naturally present in most vascular endothelial cells. Therapeutic benefits of iNOS expression in tumors using exogenous genes have previously been explored *in vitro* 26-30 and *in vivo* 31,32*.* The *in vivo* experiments highlighted the potential of sensitizing tumors through iNOS and gene transfection, but to prevent off-target effects they were achieved by infusing plasmid DNA (pDNA) directly into the tumor. Furthermore, iNOS gene therapy has not been explored in treating high-risk NB or other pediatric solid tumors. Our approach uses the targeted delivery of iNOS plasmids that are transfected into the tumor vasculature using focused ultrasound (FUS) and custom ultrasound contrast agents (UCAs) called “microbubbles”.

Microbubbles (MBs) are gaseous spheres enclosed within a phospholipid shell, the presence of which attenuates gas diffusion out of the bubble 33. They typically span 1 to 10 mm, making them smaller than the ultrasonic wavelengths used in medical imaging, and as such they serve as point scatterers rather than reflectors of ultrasound 34. Due to the compressibility of their gas cores, MBs volumetrically expand and contract in phase with external pressure changes caused by a sound wave 35. At high acoustic pressures, inertial forces triggered by MB implosion can rupture cell membranes (reversibly) and provide direct access to the endothelial cell cytoplasm 36,37. Therefore, by judiciously applying FUS, MBs' interaction with ultrasound can be spatiotemporally fine-tuned to achieve site-specific release of plasmids *in vivo*, a technique referred to as “sonoporation” or “sonopermeation”.

**Figure 1:**
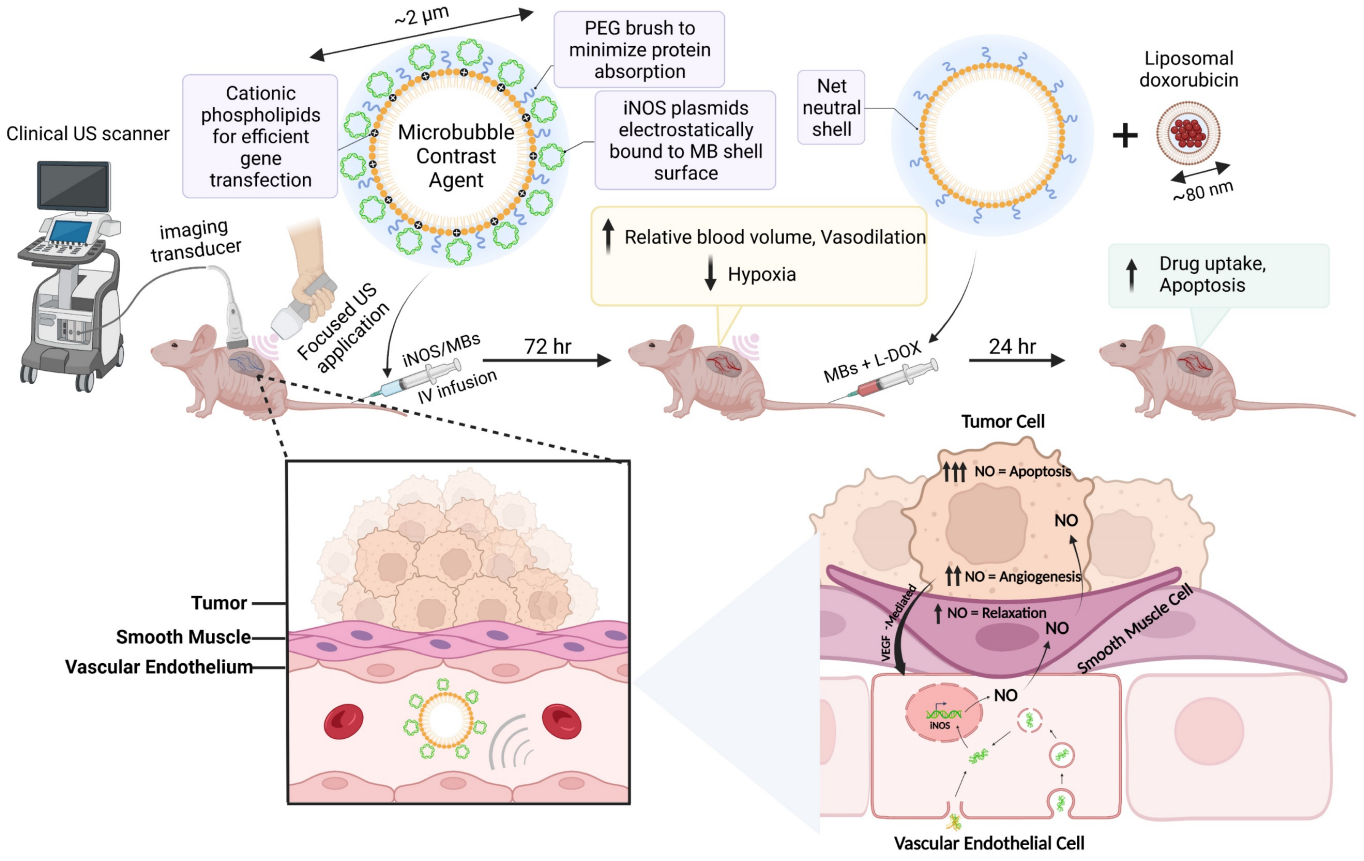
Schematic detailing the experimental procedure and the proposed mechanisms of action of iNOS gene therapy. Created with BioRender.com

Our driving hypothesis is that localized iNOS gene therapy can favorably alter the vascular properties of NB to improve tumor sensitivity to sonopermeation with liposomal nanodrugs. Following iNOS gene transfection, we capitalized on the MB's dual functionality as a theranostic tool to gauge increases in tumor perfusion stemming from raised intratumoral iNOS levels. Using long-term quantitative contrast-enhanced ultrasound (qCEUS) imaging, we monitored longitudinal changes in tumor perfusion to demonstrate the effects of iNOS gene therapy, followed by administration of liposomal doxorubicin (L-DOX) chemotherapy to increase its delivery and retention (Figure 1).

This work describes a clinically viable solution to ameliorate NB response to therapy without increasing side effects. The techniques outlined in this study (1) improve non-viral gene delivery to tumors, (2) monitor dynamic changes in the tumor vasculature in response to NO treatment, and (3) bestow control of tumor vascular properties *in vivo* while enabling real-time feedback to determine when tumors are primed for primary treatment. The results of this study demonstrate that MB-mediated non-viral transfection of vascular endothelial cells is an effective approach to enhancing tumor perfusion and liposomal drug accumulation. We anticipate that the strategy proposed in this study could have a significant impact on other pediatric and adult solid tumors as well.

## Materials and Methods

### Preparation of Microbubbles

Net neutral MBs for imaging tumor perfusion were formulated using a lipid film composed of 14.34 mg of 1,2-distearoyl-*sn*-glycero-3-phosphocholine (DSPC, 790.16 MW) and 5.66 mg of *N*-(methylpolyoxyethylene oxycarbonyl)-1,2-distearoyl-*sn*-glycero-3-phosphoethanolamine (DSPE-PEG2000, 2805.97 MW) (NOF Corporation, Tokyo, Japan), dissolved in chloroform (Sigma-Aldrich, St. Louis, MO). Cationic MBs for electrostatically binding pDNA 38,39 were likewise fabricated using a lipid film comprising 11.35 mg of DSPC, 5.76 mg of DSPE-PEG2K, and 2.88 mg of 1,2-stearoyl-3-trimethylammonium-propane (DOTAP, 702.57 MW) (Avanti Polar Lipids, Alabaster, AL). The lipid solution was evaporated for 48 h and then stored as a lipid film in a sealed scintillation vial at -20 °C. On the day of intended use, the 20 mg film was diluted to 2 mg/mL (10 mL total) in a filtered mixture of 10% propane-1,2-diol (propylene glycol, 76.1 FW) (v/v), 10% propane-1,2,3-triol (glycerol, 92.09 FW) (v/v), and 10× phosphate buffer saline (PBS) diluted to 1× (Fisher Scientific, Waltham, MA). The lipid solution was heated to 65 °C on an Isotemp Heating Block and bath sonicated in a 1.9 L Ultrasonic Bath Sonicator (Fisher Scientific, Waltham, MA) until the lipid was completely suspended. High-concentration MBs were generated using a probe micro-tip sonication method previously described by Feshitan *et al.* 40. The heated lipid solution was placed in contact with the sonicator tip (Branson 450 Ultrasonics Sonifier with microtip attachment, Emerson, St. Louis, MO) and operated at 70% power under constant flushing with Decafluorobutane (PFB, 238 MW, FluoroMed LP, Round Rock, TX) for 10 s. The combined lipid suspension was supercooled in an ice bath and then washed three times in a 10 mL Luer tip syringe (BD, Franklin Lakes, NJ) at 300 relative centrifugal force (RCF) for 3 min in a Bucket Centrifuge Model 5804R (Eppendorf, Hauppauge, NY) to collect the bubbles. The MBs were characterized using a Multisizer 4e Coulter Counter (MS4, Beckman Coulter, Brea, CA) to ensure the median bubble size was 1.90 ± 0.925 µm. The pDNA adsorption properties of the cationic MBs used in this study have been previously established in the literature 41, and estimated to be 0.05 pg/µm^2^. Furthermore, these cationic bubbles are made from material similar to commercially available lipoplexes used in non-viral gene therapy and have been extensively used and described in the literature (for recent review see 42).

### Preparation of Plasmid DNA

The mKate expression vector is from a modified pmKate2-C vector (Evrogen #FP181) that has been custom designed for integration into the rosa26 genomic safe harbor locus. The iNOS expression vector was constructed by replacing the mKate2 ORF with *M. musculus*-derived pBS-iNOS, which was a gift from Charles Lowenstein (Addgene plasmid #19295) 43. The transcript was cloned using PCR with Q5^®^ High-Fidelity DNA Polymerase (NEB, #M0492S) and primers P1: cagtagaccggtgagactctggccccacgggacacag and P2: cagtagcaattggaattgtaatacgactcactatagg. The PCR amplicon containing the iNOS transcript and the mKate2 expression vector was digested with AgeI-HF (NEB, #R3552S) and MfeI-HF (NEB, #R3589S). The iNOS transcript was cloned into the expression vector with T4 DNA Ligase (NEB, #M0202S), replacing the mKate2 ORF. Although genetic elements for homology recombination are present in both plasmids, integration was not assessed in this study, and all results are assumed to originate from the transient plasmid expression.

### Orthotopic NGP Tumor Model and Implantation

NGP cells are MYC-N amplified 44, and thus function as an appropriate model for poor prognosis NB. They reproduce many features of clinical NB, such as histology, frequency, and location of metastatic lesions when renally implanted 16, as was done in nude athymic mice (Charles River, Wilmington, MA) to generate tumor models for this study. Mice were firstly anesthetized with inhalable isoflurane. After being positioned in a sterile environment, the entire right side of each mouse was cleaned with ethanol and painted with Betadine. A 3-5 mm diagonal incision was made with a scalpel blade toward the ribcage atop the kidney. The underlying fascia was cut with scissors to expose the mouse's right kidney. A 27-gauge needle (of length 1.3 cm, BD Biosciences) fitted to a syringe containing 20 μL of cell suspension (1x10^6^ NGP cells in Phosphate Buffered Saline, Leibniz Institute DSMZ-GmbH, Braunschweig, Germany) was inserted into the kidney and its contents injected slowly. The kidney was then returned to the abdominal cavity. The fascia was closed with absorbable sutures, followed by staples to seal the skin. Mice were monitored daily to confirm complete recovery, and tumors were allowed to grow for 4-5 weeks (1-2-g weight) before ultrasound experiments were initiated.

### Mouse Preparation for Imaging and Sonopermeation

All procedures were performed in accordance with the guidelines stipulated in a protocol approved by the Institutional Animal Care and Use Committee (IACUC) at the University of Texas at Dallas. Mice were anesthetized with 1-2% isoflurane (Vedco, St. Joseph, MO) and restrained in the prone position. After confirming the depth of anesthesia by toe pinch, the animals were catheterized via either the left or right lateral tail vein using a winged butterfly infusion set (Terumo Corporation, Tokyo, Japan). Whole body temperature of the mice was maintained at 37 °C using a closed loop temperature control system comprising a heat lamp and a rectal probe (Physitemp Instruments, Clifton, NJ). Following sedation and catheterization, mice were transferred from the prep station to a custom 3D printed imaging stage, outfitted with a circulating water bath (T/Pump, Stryker, Kalamazoo, MI), for further treatment.

### 3D Volume and 2D Perfusion Imaging

3D imaging was performed, similar to 45, by mounting a linear 15L8 transducer, equipped with the Acuson Sequoia 512 ultrasonography system (Siemens Medical Solutions, Erlangen, Germany), on a stepper motor and sweeping it across the length of the tumor in 0.2 mm increments. Non-linear contrast images were acquired following a bolus injection of 1x10^7^ MBs in a total volume of 100 µL, administered via tail vein catheterization. Data were collected and subsequently analyzed using custom LabVIEW software, where tumor boundaries were manually segmented. The resulting series of 2D images was combined in ImageJ to compute volumetric measurements (B-mode) and to map the tumor vasculature (CPS mode). As described by Wei *et al.* 46, MB perfusion conforms to the equation y = A (1 - e^-βt^), where A is the relative blood volume (RBV) and β is the rate of reperfusion. Perfusion replenishment curves following a flash-destruction pulse were generated from CPS data and fitted to this form in the LabVIEW software. Quantitative measures were extracted and compared pre- (Day 0) and post-transfection (Day 3) and plotted on the same set of axes for each mouse. Statistical analysis was performed using excel using ANOVA followed by a Tukey HSD comparison between groups.

### Sonopermeation *In vivo* Using Focused Ultrasound Application

The image-guided sonopermeation procedure has been described by our group in a previous publication 45. Briefly, a custom lens and cone system was 3D printed and affixed to a therapeutic ultrasound machine (SoundCare Plus, Austin, TX) to attain a maximum pressure of ~2MPa in the focal zone. A commercial infusion pump (Kent Scientific, Torrington, CT) was coupled to a custom 3D printed rotating syringe platform, designed to evenly disperse MBs in solution, ensuring that injections were dispensed at a fixed concentration throughout the duration of MB administration. On the day of transfection (Day 0), 1x10^9^ cationic MBs were combined with 500 µg of pDNA (either mKate or iNOS) and brought up to a total volume of 500 µL with sterile saline. The MB mixture was infused into the tumor space at a constant rate of 50 µL/min and the tumors were sonopermeated by hand (3 W/cm^2^, 1 MHz, 10% duty cycle) on/off in intervals of 5 s over a period of 10 min. Post-sonopermeation, mice were checked daily to ensure that tumor burden did not exceed the euthanasia criteria (>2-g weight) delineated in our IACUC protocol and were further evaluated for any behavioral deficits related to pain or distress.

On the day of chemotherapeutic treatment (Day 3), 1x10^9^ regular MBs were combined with 1 mg/kg of L-DOX (Doxoves^©^, FormuMax, Sunnyvale, CA) and brought up to a total volume of 500 µL with sterile saline. The tumors were again hand scanned in the same manner as was done 72 h prior. Untreated controls received no gene transfection or L-DOX + sonopermeation. Untransfected tumors received L-DOX + sonopermeation only. Sham tumors received sonopermeation with non-functional pDNA and L-DOX + sonopermeation.

### Animal Survival Studies

Survival *in vivo* experiments were performed as detailed above. Briefly, mature tumors were primed with gene therapy (either mKate or iNOS), transfected by sonopermeation with 1x10^9^ cationic polydispersed MBs having median diameter ~2 mm. 72 h following gene therapy treatment, sonopermeation was performed using net-neutral MBs to deliver liposomal doxorubicin as described above. Note that for this experiment, due to the volume of bubbles needed, size-isolated microbubbles (SIMBs) were obtained from Advanced Microbubbles Inc to perform imaging and sonopermeation. Tumors were measured every other day until they reached the endpoint criteria up to 14 days using calipers, and re-imaged with a bolus of 1x10^7^ SIMBs on Day 7 as well as re-dosed with L-DOX in conjunction with sonopermeation. Normalized tumor growth over the two-week observation period was obtained by dividing the volume on any given day by initial tumor volume. Kaplan-Meier curves were generated to plot the number of days it took for tumors to increase by 50% above their starting volume up to 14 days. Survival curves were plotted, and statistical analyses were conducted in Graphpad (Prism 6). Statistical significance between groups represents Mantel-Cox test, with p < 0.05 interpreted as significant.

### Tumor Excision

Mice were sacrificed 24 h post-chemo administration (4 days following transfection) by exsanguination to eradicate the drug remaining in circulation. Before exsanguination, the mice were anesthetized using 5% isoflurane. After verifying the depth of anesthesia by toe pinch, the animals were catheterized and injected with a lectin stain (DyLight 594-LEL, Vector Laboratories). The lectin was allowed to circulate for 3 min, following which the mice were perfused by intracardiac injection of cold saline. This procedure was performed by inserting a syringe with 10 mL solution into the left ventricle of the heart and snipping the right atrium to allow blood to drain following a full flush of the mouse's circulatory system. All tumors were surgically excised for *ex vivo* processing immediately after perfusion.

### Tumoral Hypoxia Measurements

60mg/kg pimonidazole-HCl (Hypoxyprobe, Massachusetts) was injected intraperitoneally 30 min before sacrifice, and tissues were harvested and processed as described above.

### Doxorubicin Quantification in NGP Tumors

To quantify doxorubicin in excised tumors, two protocols from Bing *et al.* 47 and Head *et al.* 48 were adapted and merged. Following excision, the tumors were weighed and flash-frozen. Tissue chunks (typically 200-400 mg) were placed in 1.5 mL centrifuge tubes with a cell lysis buffer (consisting of 0.25 M sucrose, 5 mM Tris-HCl, 1 mM MgSO_4_, 1 mM CaCl_2_ pH 7.6) and 100 µL ceramic beads (MO BIO Laboratories, Carlsbad, CA). The tubes were vortexed (Bristol-Meyers Squibb, New York, NY) for 45 s to homogenize the tissue. To establish standards of known doxorubicin measurements in homogenates of tumors, untreated tissue was mixed with 2 µL of 10 mg/mL doxorubicin (Sigma Aldrich, St. Louis, MO) stock dissolved in DMSO and homogenized as described above. Spiked homogenates were then serially diluted with extraction buffer. The readings of untreated tumor samples without doxorubicin were considered zero. Untreated and treated homogenized samples (200 µL) were placed in microcentrifuge tubes with acidified isopropanol solution: 100 µL of 10% (v/v) Triton X-100 (Sigma Aldrich), 200 µL of water, and 1 mL of acidified isopropanol (0.75-N HCl, Sigma Aldrich). Samples were stored overnight at -20 ºC to extract the doxorubicin. The next day, the tubes were warmed to room temperature, vortexed for 45 s, centrifuged at 2,000 g for 15 min and stored at -80 ºC until analysis. A five-point standard curve generated by spiking tissue with known quantities of doxorubicin was run side by side with experimental samples to quantify the uptake per gram of tumor using linear regression. Statistical analysis was performed using excel using ANOVA followed by a Tukey HSD comparison between groups.

### Immunohistochemistry

Excised NGP tumors were embedded in Tissue-Tek® optimum cutting temperature (O.C.T.) compound (Electron Microscopy Services), then stored at -20 ºC until cryosectioned (Leica CM1860). 15 μm thick cryosections were fixed with acetone and permeabilized with Tween 20. After blocking non-specific binding with CAS-Block Histochemical Reagent (ThermoFisher Scientific), the following primary antibodies were used: murine iNOS (1:500, #13120, Cell Signaling), aSMA-Cy3 (1:1000, #C6198, Sigma), and pimonidazole (1:100, #Pab2627, Omnikit, Hypoxyprobe, Massachusetts). Isolectin-B4-AF568 (1:100, #I21412, Invitrogen) was diluted in HEPES buffer. Alexa Fluor 488 goat anti-rabbit IgG (Invitrogen) secondary antibody was applied following incubation in primary solution and a series of washing steps. Finally, the slides were mounted with DAPI (VECTASHIELD PLUS Antifade Mounting Medium with DAPI, Vector Laboratories).

### Quantitative *Ex Vivo* Imaging

For iNOS expression colocalization studies, sections were imaged on a Marianas Confocal (Zeiss) using a 40X oil objective, capturing 14 steps of 0.33 μm on the z-axis, with a resolution of 0.33 μm per pixel. At least five images of each tumor were taken, with four tumors per group. To avoid bias, the endothelial marker Isolectin-B4 was used to determine image capture and focus and iNOS staining was captured at equal exposure times for all tissues. The images were then analyzed in ImageJ (NIH, USA), selecting the lectin-positive area of the picture taken to create the area to be quantified. This area was then used to quantify the mean intensity of the iNOS staining within the tumor endothelium. The mean iNOS intensity within each tumor endothelium z-stack was then averaged to obtain the mean iNOS intensity of each tumor blood vessel analyzed. Averages were then obtained for each tumor. Because tumor cells were injected directly into the kidney and often coopt glomeruli and tubules 49, we quantified iNOS expression in the adjacent kidney in a separate analysis, following the same method as tumor iNOS quantification. Quantification of pimonidazole and Isolectin was performed using the average intensities from the respective stains comparing the iNOS treated and Sham groups using FIJI (NIH). Terminal deoxynucleotidyl transferase dUTP nick end labeling (TUNEL) staining was performed on fresh frozen sections following the manufacturer's instructions (Millipore, USA), and was visualized on a whole slide scanner (Olympus VS120 Virtual Slide Microscope) at 447 nm (DAPI), 510 nm (FITC), and 624 nm (Texas Red) laser wavelengths.

The distance of aSMA from lectin was measured using the distance tool on scans using the Olympus VS120 software tools. 25 measurements were taken from each tissue section and the averages of three sections per group were then used to calculate statistical differences using Student t-test (GraphPad Prism), with the significance threshold set at p ≤ 0.05.

## Results

### Low-Dose L-DOX Synergizes with iNOS Overexpression in NGP Cells *In Vitro*

**Figure 2:**
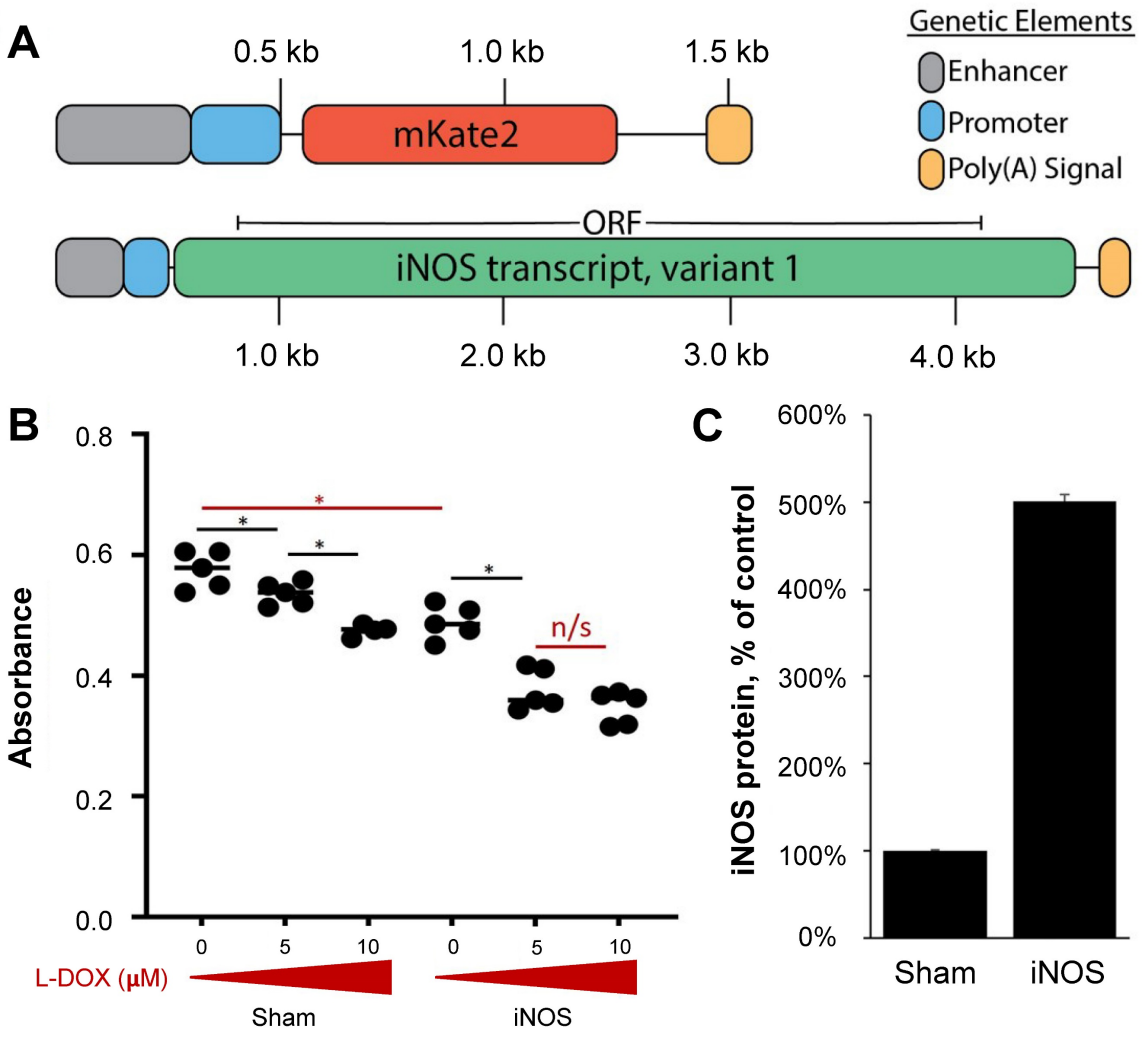
iNOS and Sham plasmids interact with L-DOX in NGP tumor cells. (A) Sequence maps of mKate and iNOS plasmids, which were deposited to the NGP tumoral vascular endothelium via MB-mediated transfection. Negatively charged pDNA electrostatically binds to cationic bubbles when commingled; this complexation shields the genetic material from being degraded in circulation. Enhancer and promoter elements are derived from CMV, and the poly(A) signals are derived from SV40 virus. (B) *In vitro* NGP cells transfected with Sham or iNOS followed by increasing L-DOX concentrations demonstrate that iNOS decreases cell proliferation compared to Sham in the absence of L-DOX (red bar), and that low dose L-DOX synergizes with iNOS to further decrease NGP cell proliferation (n = 5 per group). (C) Protein extracts interrogated with ELISA harvested from parallel experiments indicate that iNOS transfection leads to a 5-fold iNOS upregulation compared to Sham (n = 3 per group).

Multiple studies have shown that iNOS alone can have adverse effects on multiple tumor cells *in vitro* 50-52. To evaluate the iNOS interaction with L-DOX in NGP cells, we assessed NGP cell proliferation after transient iNOS transfection with increasing L-DOX concentrations (0, 5 and 10 mM). In the absence of drug, iNOS transfection alone reduced proliferation by 15% compared to Sham (Figure 2B, Sham 0.57 ± 0.003 *vs.* 0.49±0.005 iNOS, absorbance units, p < 0.01). In the Sham group, low L-DOX concentrations (5 mM) resulted in a significant albeit small (7%) reduction in cell numbers compared to no L-DOX (Figure 2B). In comparison, iNOS sensitized NGP cells to 5 mM L-DOX with a 22% reduction in cell number relative to untreated iNOS cells (in iNOS cells, 0.49 ± 0.03 no L-DOX *vs.* 0.38 ± 0.03 low L-DOX, absorbance units, p < 0.001). This interaction did not extend to higher L-DOX doses, however, as iNOS-transfected cells receiving either 5 or 10 mM L-DOX were not different from each other (0.38 ± 0.03 low L-DOX *vs.* 0.35 ± 0.035 high L-DOX, absorbance units, p = 0.17). This suggests that iNOS sensitizes NGP cells to L-DOX only in lower L-DOX concentrations. Protein from NGP cells transfected and harvested at the time of cell proliferation assay indicate that iNOS transfection resulted in a 5-fold iNOS overexpression compared to Sham (Figure 2C, 0.04 units Sham *vs.* 0.2 ± 0.03 units iNOS, n = 3, p < 0.0001). Together, this data shows that iNOS overexpression inhibits NGP cell proliferation by 15%, and that its interaction with L-DOX accounts for a further 12% cell loss for a combined 27% reduction in cell proliferation.

### Sonopermeation Using Focused Ultrasound and Cationic Bubbles Specifically Transfects Tumor Vascular Endothelium

**Figure 3:**
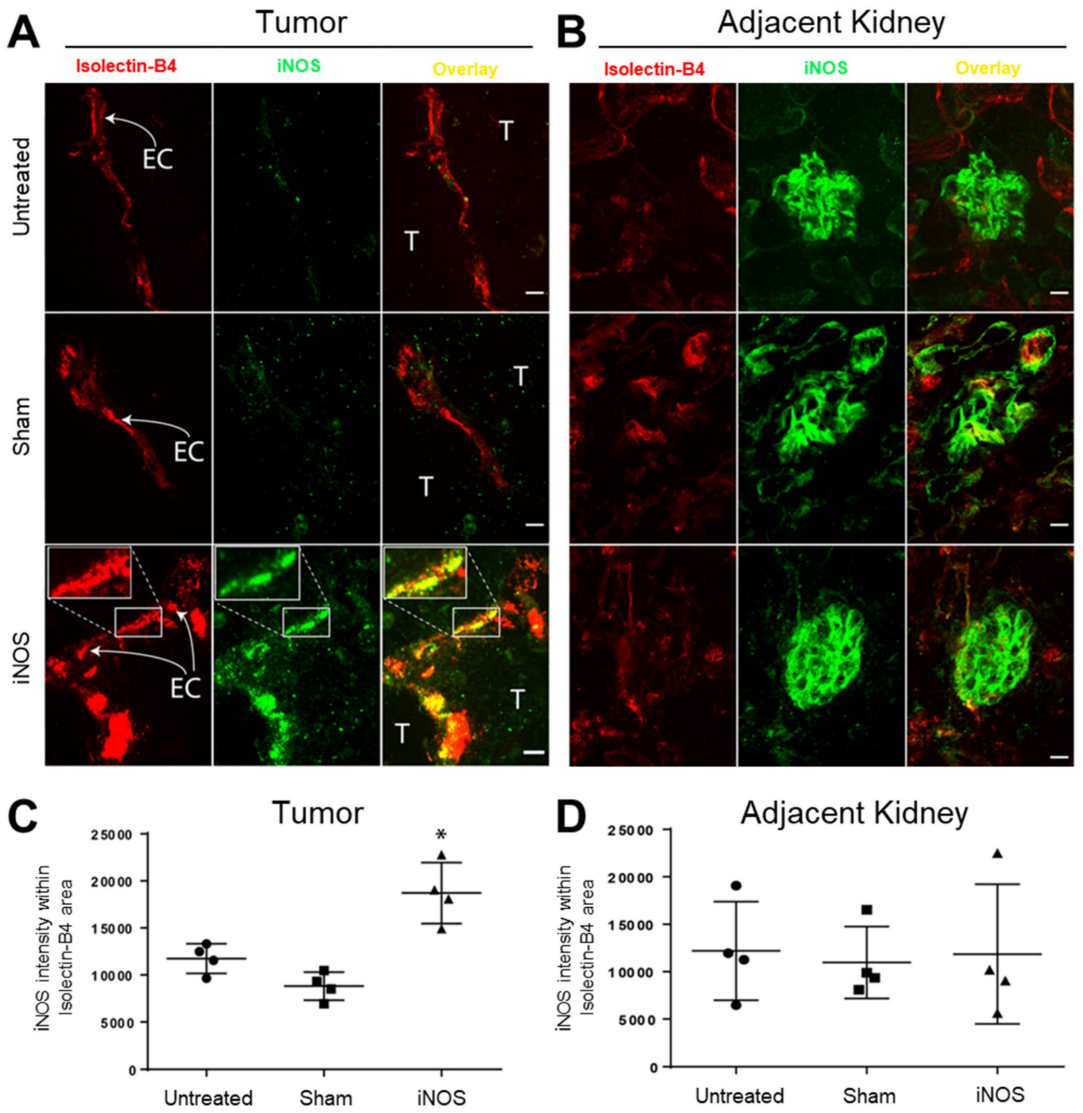
iNOS transfection via sonopermeation increases iNOS expression in tumor endothelial cells in NGP tumor-bearing mice but not in adjacent kidney structures. (A) Confocal microscopy images reveal elevated iNOS expression in tumor endothelium after iNOS transfection compared to untreated or Sham-transfected tumors (yellow overlay in bottom right panel and inset). EC: endothelial cells, T: tumor cells. (B) Representative images of glomeruli (labeled “G”) adjacent to NGP tumors. (C) Quantification of the iNOS signal intensity shows a greater than 2-fold increase within the lectin-positive area in iNOS-transfected tumors compared to untreated or Sham-transfected tumors, indicating enhanced iNOS activity. (D) iNOS expression in adjacent glomeruli remains unchanged after iNOS or Sham transfections (p > 0.05). Scale bar = 10 µm. n = 4 per group.

As mice were perfused with the endothelial marker Isolectin-B4 prior to euthanasia, the presence of this stain in sections represents endothelial cells of functional vasculature. We evaluated iNOS expression in the tumor vasculature using Isolectin-B4 72 h after transfection to allow for maximal protein expression. Figure 3A highlights representative images of tumors while Figure 3B highlights representative images from the adjacent non-targeted healthy kidney tissue; both sets of images were derived from projections of z-stacks obtained with confocal microscopy. Endothelial cells are labeled “EC” in the figure and adjacent tumor cells are marked “T”. Immunohistochemistry analysis revealed a significant increase in endothelial iNOS expression in the iNOS-transfected endothelial cells (Figure 3A, lower middle and right panels, yellow overlap), while Sham-transfected tumor endothelial cells had no change in iNOS compared with untreated tumors (Figure 3A, middle row shows no increase in iNOS, green, compared to untreated controls). A 3D animation in Supplementary Video 1 highlights the overlay of Isolectin-B4 in red, and iNOS in green, resulting in overlapping yellow in a transfected tumor blood vessel. To quantify the iNOS expression in the tumor endothelium, we restricted iNOS intensity within the lectin-positive areas (Figure 3C). Quantification of the iNOS mean intensity within the lectin-positive area revealed over 2-fold increase in iNOS expression after iNOS transfection compared to Sham transfected tumors and over 1.5-fold compared to untreated controls (Figure 3B, mean intensity values: 11,749 ± 1,576 untreated, 8,814 ± 2,952 Sham, 18,709 ± 5,993 iNOS, p < 0.01). To evaluate the specificity of iNOS sonopermeation, we quantified iNOS expression in neighboring kidney structures (Figure 3C, glomeruli labeled “G”) using the same methodology used to quantify tumor iNOS expression. In the adjacent kidney, we found no change in iNOS expression regardless of the group, suggesting that iNOS overexpression was localized to the targeted sonopermeated tumor.

### Transfection with iNOS Increases Blood Volume and Flow in Neuroblastoma Xenografts

**Figure 4:**
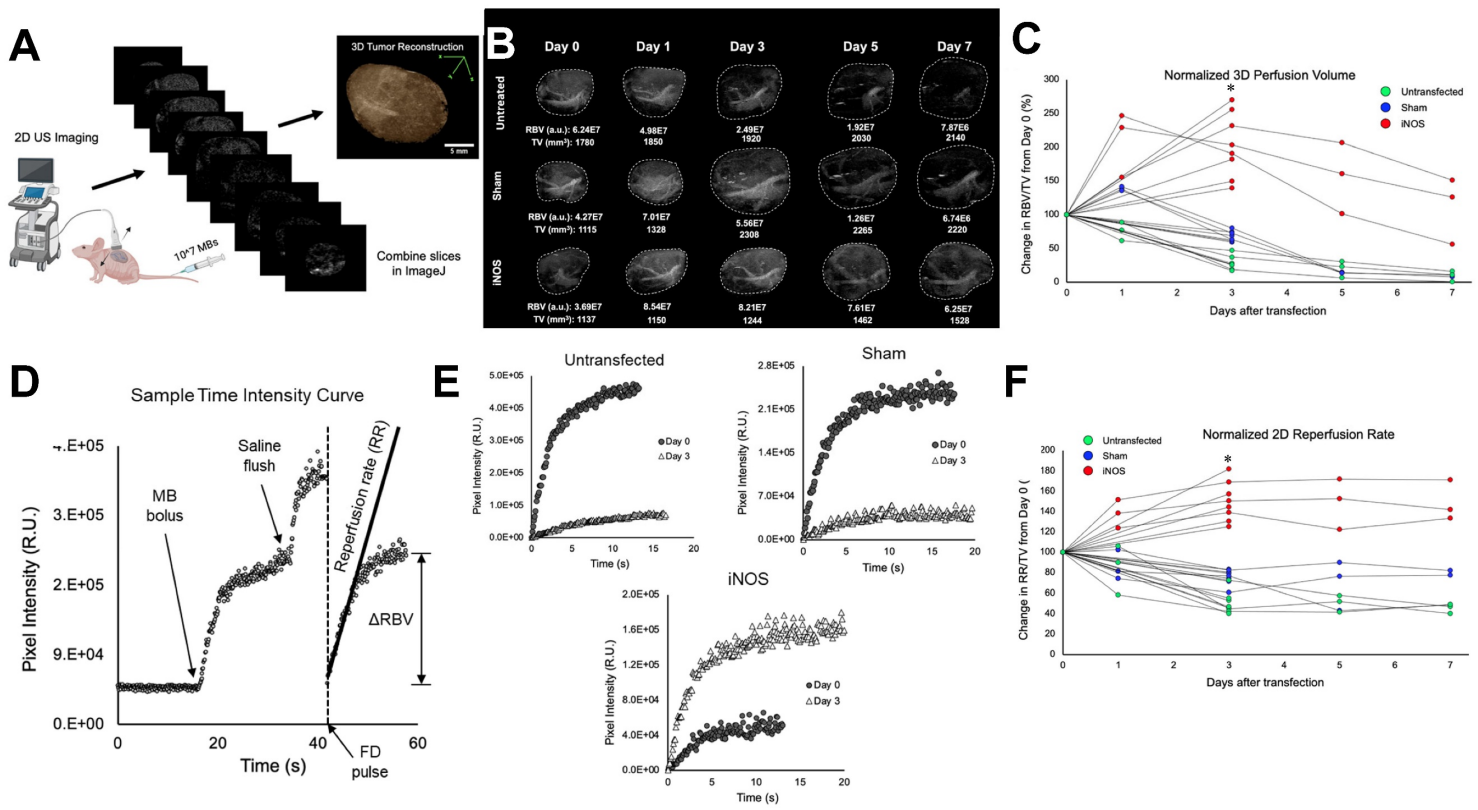
Longitudinal effects of iNOS transfection on NGP tumor vasculature and perfusion dynamics in a 7-day time course. (A) Schematic representation of the workflow for generating 3D tumor reconstructions from 2D contrast images. (B) Representative 3D contrast images displaying the increase in perfusion-volume post-treatment in comparison with Sham-treated and untreated controls. (C) Normalized tumor perfusion volumes are plotted over a 7-day period, demonstrating a significant increase in perfusion post-treatment. (D) Time-intensity curves are derived from non-linear 2D imaging to assess perfusion dynamics in NB tumors. (E) Flash-destruction (FD) replenishment curves reveal augmented perfusion rates in iNOS-treated tumors compared to controls. (F) Quantitative analysis of normalized tumor 2D re-perfusion rates after FD indicates enhanced perfusion in response to iNOS treatment over the 7-day time course. n = 7-8 mice per group. * indicates p < 0.05.

Quantitative contrast-enhanced imaging was performed at 0 (before transfection) and 3 days post transfection. Several mice from each treatment group were also monitored on days 1, 5, and 7. Perfusion volume was gleaned from whole tumor 3D reconstructions (Figure 4A) and flow rates were extracted from 2D MB time-intensity curves (TICs) following a flash-destruction pulse (Figure 4D). Blood perfusion volume (RBV) was determined in LabVIEW by summing pixel intensities throughout the tumor volume following an intravenous injection of MBs. Since MBs rapidly mix and circulate with blood upon systemic infusion, the overall enhancement in video signal intensity denotes the total blood pool volume in that region 53. Examples showing 0-3 day and 0-7 day perfusion trends are displayed in Figure 4B. To generate a measure of the density of vessels within the tumor, RBV values were divided by tumor volume (TV) and plotted during the week after transfection (Figure 4C). Vascularity changes were seen as early as 24 h and the change in RBV from baseline was calculated at 3 days post-transfection. iNOS tumors increased in vascularity by 213 ± 47% while Sham and untreated controls decreased to 68 ± 7% and 32 ± 12% respectively. ANOVA followed by the Tukey HSD test was performed. The p-value corresponding to the F-statistic of one-way ANOVA was lower than 0.05, suggesting that the one or more treatments are significantly different. The Tukey HSD p-values for the iNOS group vs. the Sham and untreated control groups were p < 0.01. The p-value between the Sham and untreated control was p = 0.06, indicating that these groups were not statistically different on day 3. Interestingly, the vascularity of the Sham group did appear to increase at 24 h, consistent with our previous findings reported in Theranostics 45, supporting the conclusion that sonopermeation increases vascular permeability and enlarges vessel lumens (reversibly) for up to one day post-treatment.

The rate of MB reperfusion (RR), which is representative of blood flow, was normalized to TV and monitored from 0-3 days or over a one-week period (Figure 4F). Flow rates improved drastically 3 days after iNOS treatment (150 ± 18%) and remained at increased levels over the next few days. Sham-treated mice and untreated control mice both showed lower reperfusion at day 3 (77 ± 8% and 50 ± 10% respectively) and continued to decrease over 7 days. ANOVA followed by the Tukey HSD test was performed. The p-value corresponding to the F-statistic of one-way ANOVA was lower than 0.05, suggesting that the one or more treatments are significantly different. The Tukey HSD p-values for the iNOS group vs. the sham and untreated control groups were p < 0.01 and the Sham vs. untreated control group had a p < 0.01, indicating that all groups were significantly different.

### Transfection with iNOS Increases Perfusion in Neuroblastoma

**Figure 5:**
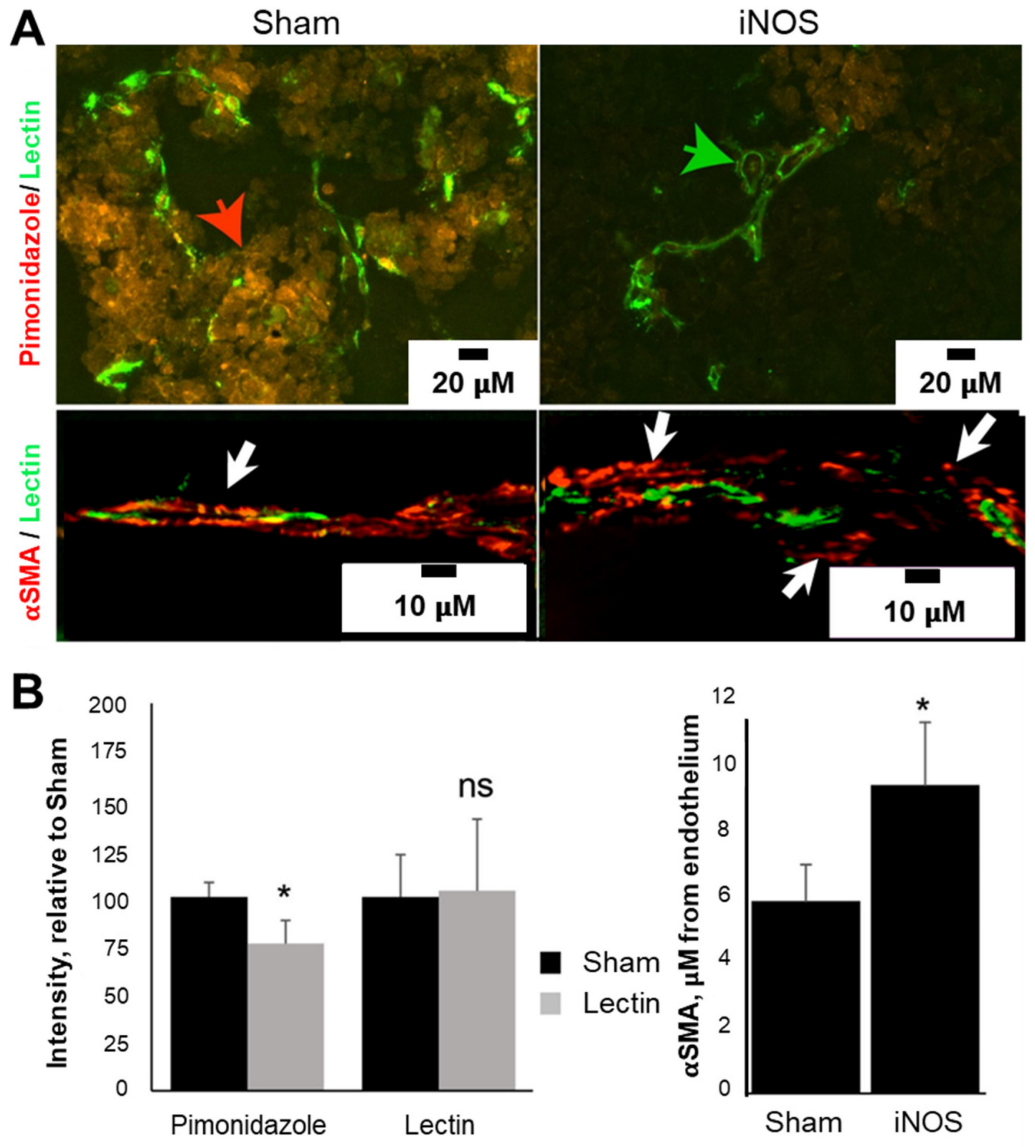
iNOS transfection increases tumoral perfusion by remodeling tumor vasculature and reducing tumor hypoxia. (A) iNOS transfection reduced tumor hypoxia as indicated by a decrease in pimonidazole immunoreactivity (red arrow) relative to Sham-transfected tumors. Increased vascular diameter or lumens (green arrow) was evident in iNOS tumors. αSMA-positive pericytes were located further away from lectin-positive endothelial cells in iNOS-transfected tumors (white arrows), than Sham tumors (yellow arrow) with adjacent or overlapping pericyte coverage of endothelium. (B) Quantification of pimonidazole and lectin stains (left) demonstrates decreased hypoxia in iNOS tumors. Pericyte distance from the endothelium increased as a result of iNOS transfection (right). n = 5 mice per group. * indicates statistical significance (p < 0.05) in the amount of hypoxia and pericyte quantifications.

Changes in vascularity post-transfection were interrogated using immunohistochemical analysis on tumor tissues harvested 72 h after transfection. The endothelial marker, Isolectin-B4, revealed no change in the total amount of endothelial cells, but an increase in the vascular lumen was evident, consistent with NO vasodilation effects (Figure 5A, green arrow), suggesting that angiogenesis is not occurring because of iNOS transfection. In line with the pericyte relaxation effects of NO, the distance between alpha smooth muscle actin (aSMA) pericytes and endothelium was 60% higher in iNOS-transfected tumors compared to the Sham group (Figure 5B, 5.8 ± 1.1 mm Sham vs. 9.3 ± 1.9 mm iNOS, p < 0.05). An example of pericyte distance from endothelium is depicted in Figure 5A (lower panel, white arrows), unlike Sham tumors where pericytes are either directly adjacent or overlap with the endothelium (left panel, yellow overlay, white arrow). To gauge hypoxia, we quantified pimonidazole injected 30 min before sacrifice; staining revealed a 24 ± 12% (p < 0.05) decrease in the amount of pimonidazole in the iNOS-transfected tumors compared to the Sham group (Figure 5A, red arrow and Figure 5B, graph). Together, these findings demonstrate a sustained tumoral vasodilation that increases blood volume, along with decreased pericyte support of endothelial cells resulting in increased permeability and reduced intratumoral hypoxia levels.

### iNOS Transfection Increases Doxorubicin Uptake in Orthotopic NB Xenograft Tumors and Increases Apoptosis

**Figure 6:**
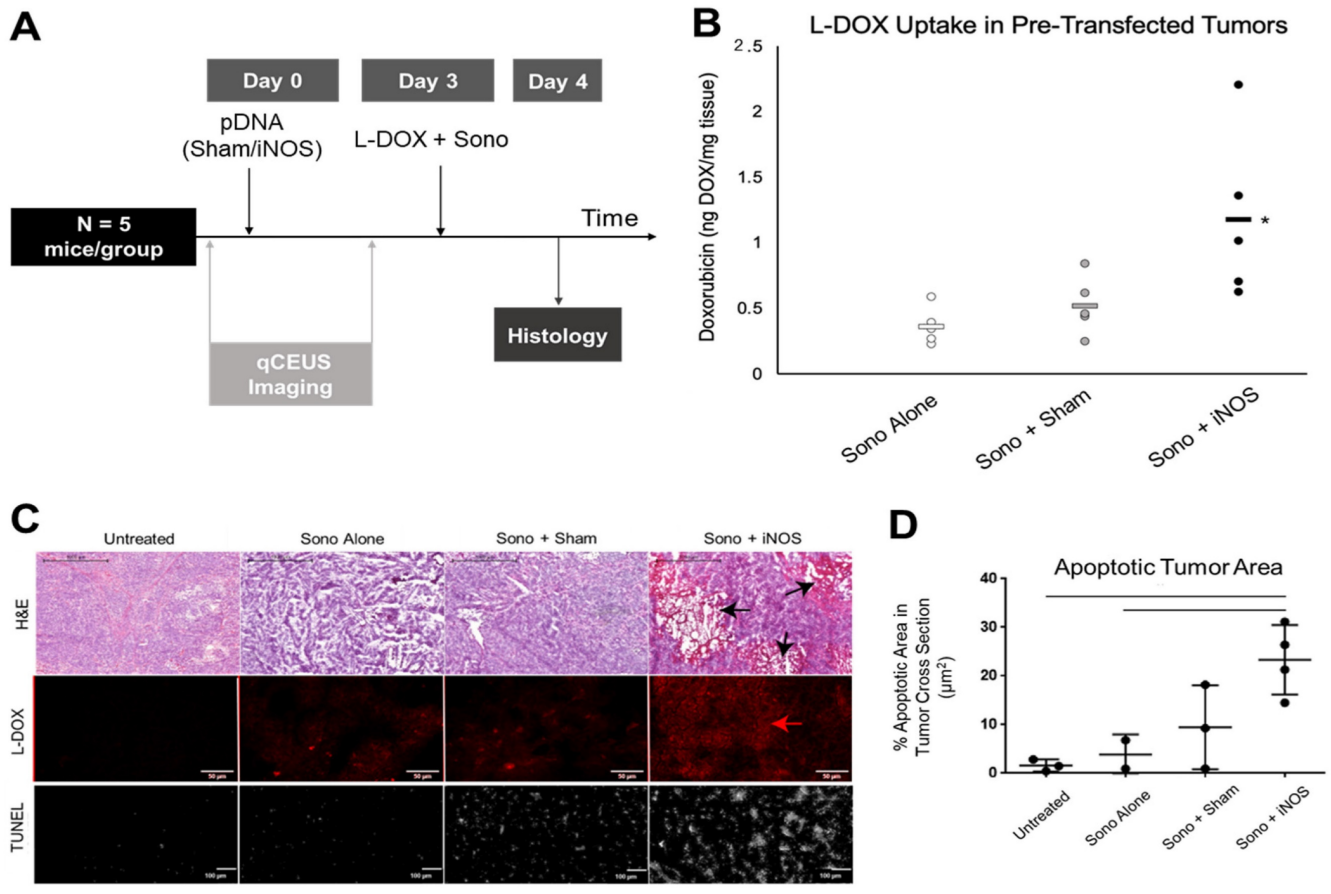
Effect of pre-treatment with iNOS gene therapy on doxorubicin uptake. (A) Summary of the methodology for priming tumors with iNOS before treatment. qCEUS imaging was performed using a bolus of 1x10^7^ regular MBs before sonopermeating on days 0 and 3. Tumors were excised and processed for *ex vivo* analysis 24 h post-chemo. (B) Pretreatment of tumors with iNOS significantly increased drug uptake, as demonstrated by a 3.3-fold increase in doxorubicin accumulation compared to untransfected counterparts, and 2.3-fold boost compared to tumors receiving Sham treatment. (C) Representative images of doxorubicin uptake reveal that Sham tumors had slightly higher uptake compared to controls, while iNOS tumors had significantly higher uptake than both groups (top right panel). Similarly, examination of the apoptosis marker TUNEL showed increased staining in proportion to doxorubicin uptake, with iNOS transfection resulting in the largest areas of TUNEL staining (bottom right panel). (D) Quantification of TUNEL areas normalized by total tumor area showed that iNOS-transfected tumors had significantly higher levels of apoptosis compared to untreated controls receiving L-DOX without sonopermeation or untransfected tumors given L-DOX with sonopermeation (p < 0.05). Furthermore, this level approached significance compared to Sham tumors (p = 0.08). n = 5 mice per group in doxorubicin extraction and n = 3-4 in histological analysis. * indicates statistical significance (p < 0.05) with respect to all other groups.

To investigate whether priming with iNOS expression enhances chemotherapeutic uptake, NGP tumors were sonopermeated with 10^9^ net-neutral MBs together with 1 mg/kg L-DOX 3 days post-transfection (Figure 6A). Tumors pretreated with iNOS plasmids before L-DOX sonopermeation had a 327 ± 64% increase in doxorubicin fluorescent intensity compared to untransfected tumors receiving L-DOX sonopermeation and 228 ± 39% intensity compared to tumors pretreated with Sham plasmid (Figure 6B). Untreated controls (no sonopermeation, L-DOX alone) were used to measure the baseline fluorescence intensity for comparison. The p-value corresponding to the F-statistic of one-way ANOVA was lower than 0.05, suggesting that the one or more treatments are significantly different. The Tukey HSD test comparing the treatment groups showed that the iNOS was significantly higher than both the sham and untreated control groups (p < 0.05), but tumors pre-treated with Sham plasmids and untransfected tumors receiving L-DOX sonopermeation were not statistically different (p = 0.82) These changes in doxorubicin quantification were mirrored qualitatively by microscopy (Figure 6C, top panels, red). Finally, we quantified the percentage of areas positive for the apoptosis marker TUNEL and confirmed a higher degree of apoptosis in L-DOX sonopermeated tumors pretreated with iNOS compared to untreated controls receiving L-DOX without sonopermeation (p < 0.01) and untransfected tumors sonopermeated with L-DOX (p = 0.02) (Figure 6C, lower panel, and 6D quantification). Although the apoptosis area in the Sham group was not significantly different from iNOS tumors (p = 0.08), iNOS pretreated tumors had large non-viable areas that likely led to the undercounting of apoptosis in this group only. Jointly, these data suggest that pre-treatment with iNOS augments tumoral perfusion to the extent that higher drug payloads amass in the tumor, even when L-DOX is administered at very low doses.

### iNOS Transfection Followed By L-DOX Increases Median Survival Time

**Figure 7:**
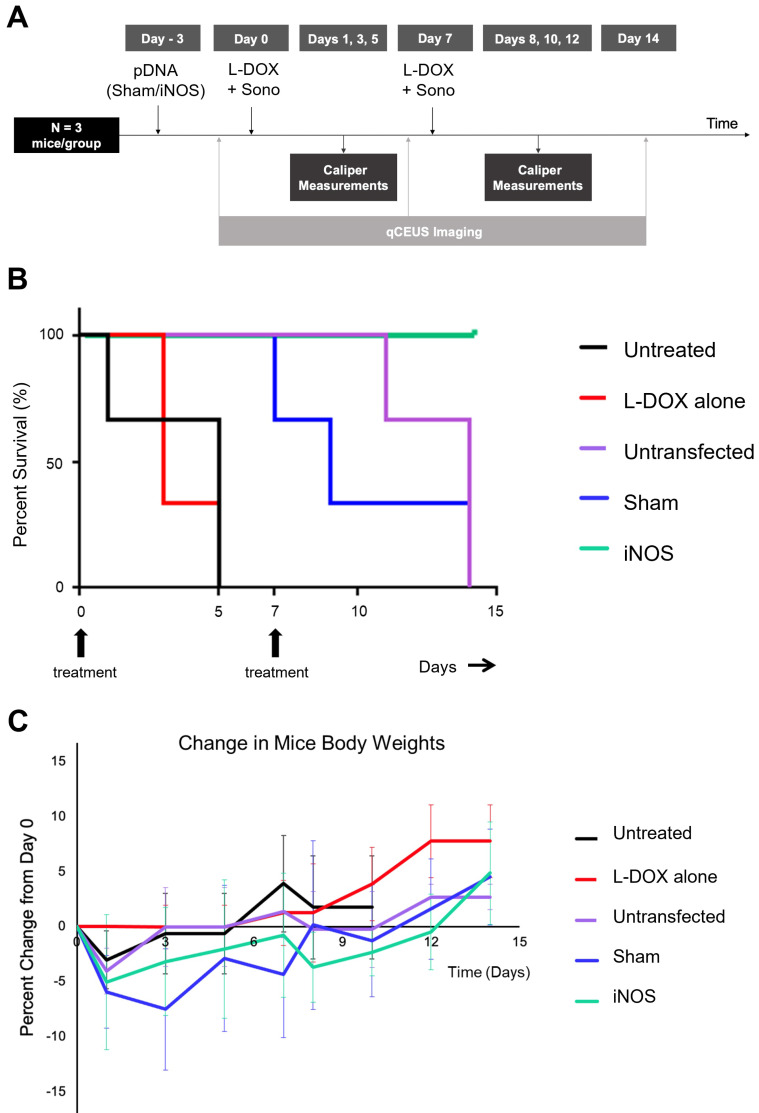
Effect of pre-treatment with iNOS gene therapy on tumor growth. (A) Summary of procedure adopted to prime tumors with gene therapy before low dosage chemotherapy plus sonopermeation; mice were re-dosed at day 7 and monitored for 14 days or until the endpoint criteria were met. qCEUS imaging was performed using a bolus of 1x10^7^ size-isolated MBs before sonopermeating on days 0, 7, and 14. (B) iNOS transfection significantly increases survival time in NGP tumors. (C) The change in percentage body weight for each treatment group is shown. n = 3 mice per group.

To understand the relationship between iNOS-mediated perfusion increases and volumetric tumor growth, two-week studies in which mice were dosed and re-dosed with L-DOX along with sonopermeation on days 0 and 7 were undertaken with and without pre-transfection. Mice with no treatment had a median survival time of 5 days (Figure 7, black line), while mice receiving only L-DOX took a median of 3 days to increase in volume by 50% above baseline (Figure 7, red line) (p = ns). In contrast, sonopermeation alone (Figure 7B, purple line) followed by L-DOX resulted in a median survival time of 14 days, while Sham plasmid transfection (Figure 7B, blue line) followed by L-DOX resulted in a median survival time of 9 days. While Sham and sonopermeation alone were not statistically different from each other (p = ns), both took significantly longer to reach 50% growth than untreated and L-DOX only mice (p < 0.05). Finally, none of the mice receiving iNOS transfection followed by L-DOX reached 50% tumor growth after 14 days, defined as the endpoint of the study (Figure 7, green line), thus surviving significantly longer than mice in any other group (p < 0.05). This longitudinal data shows that iNOS transfection combined with L-DOX treatment prolongs survival time.

## Discussion

For the past three decades, passive targeting by the Enhanced Permeability and Retention (EPR) effect has had uneven success by making use of nanoscale vehicles, such as liposomes, to shuttle cargo into tumors 54,55. While it can be effective (particularly for long-circulating liposomes), mounting evidence alludes to its highly variable nature 56, rather than a generalizable phenomenon as it was once regarded 57,58. Moreover, NB tends to be poorly vascularized, and in many cases even coopts vessels from nearby healthy tissues, which are not inherently leaky 59. The lack of fenestration and poor perfusion can be significant obstacles to drug penetration in tumor therapy.

A key conclusion from our previous work exploring image-guided drug delivery (IGDD) using sonopermeation 45 provided the impetus for this study: tumor perfusion volume serves as a robust predictor of drug accumulation. We found that doxorubicin uptake in sonopermeated tumors correlates positively with perfusion volume, indicating that the initial degree of vascularity greatly influences the extent to which sonopermeation enhances drug uptake. In prior studies, we did not control tumor perfusion, but advocated methods of transiently improving blood flow, which we argued would lead to more effective therapy. In this work, we hypothesized that pursuing NO-based gene therapy would improve tumor perfusion and allow for increased drug penetration. This is the first study we are aware of that employs a targeted non-viral gene therapy strategy to modify NB biology to improve the efficacy of a chemotherapeutic drug.

Non-viral therapies are well known for having low transgene production compared to viral vectors 60,61. Our data demonstrates that we successfully transfected tumors *in vivo* using sonopermeation, and that we increased iNOS expression in the targeted tumor vasculature (Figure 3). However, we reasoned that high levels of transgene expression may not be required for effective NO therapy when using an efficient producer of this molecule. Our paradigm therefore hinges on the delivery of a pDNA encoding a functional copy of the inducible variant of NOS, so that NO can be produced by catalytic conversion of L-arginine, independent of calcium/calmodulin signaling. Multiple isoforms of NOS exist throughout the body with varying levels of NO production. iNOS is the most potent isoform that can generate several orders of magnitude more NO (100 to 1000-fold greater) than constitutive endothelial or neuronal NOS (eNOS and nNOS), until substrate availability becomes rate-limiting 30. Several studies have highlighted the potential role of iNOS in cancer therapy 62-66. Our approach leveraged selective iNOS transfection of the tumor vascular endothelium, thus narrowing its effect on the tumor vasculature and limiting its potential effects on tumor cells. Thus, we have exploited iNOS as a means of modulating intratumoral vascular NO production, thereby enhancing tumor perfusion spatiotemporally.

This research also advances gene therapy efficacy with the use of a platform that merges ultrasound-triggered cell-entry and enhanced gene transfection. The status quo as it pertains to gene delivery is that non-viral vectors have not been widely adopted to address major *in vivo* barriers: (1) degradation of the therapeutic genes by endonucleases in the serum, (2) selective deposition of therapeutic genes into the tumor tissue, and (3) plasmid internalization into tumor or tumor vascular cells. By incorporating 20% molar mass of the cationic phospholipid DOTAP into their shell, MBs are capable of electrostatically binding negatively charged plasmids and protecting them from degradation in circulation 67. When we locally transfected orthotopic NGP xenografts by applying FUS during a systemic administration of pDNA-bound bubbles, we detected iNOS post-transfection by immunostaining, with high levels of the expressed protein product (iNOS) present 72 h after transfection (Figure 3A). iNOS overexpression within the tumor required functional iNOS -expressing plasmids (Figure 3B). Our earlier research demonstrated that gene expression occurs only in areas where FUS is applied 68, therefore effects of systemic treatment can be confined strictly to tumor tissue. These data suggest that sonopermeation is an efficient alternative to virus-mediated delivery, which is the current gold standard for gene therapy.

When we investigated whether whole tumor perfusion was affected by iNOS overexpression, we found that perfusion decreased at 3 days in untreated controls and in tumors transfected with an identical plasmid expressing a non-functional protein, mKate (“Sham”), as compared with those transfected with iNOS in which perfusion volume increased significantly (Figure 4). The sample 3D tumor reconstructions showcased in Figure 4B exemplify the global changes that are encapsulated graphically in Figure 4C: hypoxic pockets adjacent to the tumor vasculature exist in the untransfected tumors, suggesting nonfunctional vasculature, while such unperfused regions are less pervasive in the iNOS tumors. In contrast, tumors receiving iNOS transfection showed significant increases in perfusion volume within the tumor space over time (Figure 4C). This suggests that iNOS may be responsible for pericyte relaxation, as suggested in Figure 5A, thus promoting vessel enlargement. The precise explanation behind this phenomenon is still unknown; however, it could be due to oxygen redistribution towards non-respiratory targets upon intracellular elevation of NO 69, preventing cells from registering hypoxia. Hagen *et al.* conjectured that NO-dependent diversion of O_2_ reactivates enzymes whose functionalities are diminished by hypoxia, signifying that treatment with iNOS may delay the emergence of hypoxic conditions within tumors. Indeed, the hypoxia marker pimonidazole revealed iNOS expression decreased hypoxia compared to Sham (Figure 5A). Analysis of the vasculature showed widened vascular lumens and increased pericyte distance from the lumen brought on by iNOS expression, in agreement with its known effect as a vasodilator and its effects on pericyte relaxation.

The biological effects of NO therapy can be highly variable and are highly dependent on local NO concentration in tissue. It was not feasible to monitor NO concentrations directly since its half-life *in vivo* is <2 ms 70. Consequently, it was critical to observe the effects of NO therapy in real-time to identify when tumors became the most susceptible to treatment. Examining qCEUS parameters offers evidence of iNOS' vascular effects: whereas untransfected and Sham tumors experience a decrease in MB flow rates (Figure 4F), iNOS tumors show increases in both the rate of reperfusion (RR) and relative blood volume (RBV). In Figure 4E, superimposed pre- and post-transfection reperfusion curves reveal that declining RR and RBV trends in the Sham and untreated control groups are entirely reversed in the iNOS treatment group; *ex vivo* analysis of these tumors divulges that iNOS tumors present with significantly dilated vessel lumens (Figure 5). Monitoring of the tumors post-transfection using qCEUS imaging can therefore be used to identify optimal windows of therapy for treatment in a clinical setting.

Doxorubicin is an integral part of NB standard-of-care. However, cardiotoxicity associated with high dosage chemotherapy is a significant clinical problem. Our previous work has demonstrated that L-DOX can be efficiently delivered using sonopermeation. This form of doxorubicin, which is clinically available, has a much longer circulation lifetime and reduced cardiotoxicity profile compared to unencapsulated doxorubicin 71. We therefore elected to use the same strategy to test the effects of iNOS gene therapy to prime tumors for liposomal drug treatments. We administered L-DOX along with sonopermeation 72 h after iNOS or Sham transfection and quantified amplified drug accumulation in iNOS-expressing tumors *ex vivo*, both by tissue extraction and histology (Figure 6). The increase in tumoral L-DOX uptake was confirmed by DOX detection in tumoral tissue samples and a corresponding increase in tumor apoptosis quantification (Figure 6). Tumor integrity was severely compromised in several of the iNOS-transfected tumors treated with L-DOX and sonopermeation, which likely led to an underestimation of tumor apoptosis in iNOS-transfected tumors during quantification, further strengthening our findings. Heightened L-DOX uptake and apoptosis in iNOS-transfected tumors (Figure 6) also correlated with increased tumoral survival time (Figure 7). In fact, all the mice receiving iNOS transfection and low-dose L-DOX had no tumor growth at the end of the study, supporting the efficacy of this approach.

To parse out the contribution of iNOS expressed in tumor cells versus vasculature and its potential interaction with L-DOX, we overexpressed iNOS in cultured tumor cells and added increasing L-DOX concentrations. Overexpressing iNOS inhibited tumor cell proliferation and synergized with lower L-DOX concentrations (Figure 2). This interaction, however, was not observed with higher L-DOX concentrations. Our data is consistent with previous studies demonstrating that iNOS enhances cancer cell chemotoxicity 26 and radiotoxicity 72 *in vitro* and *in vivo*. Possible mechanisms for L-DOX interaction with iNOS include reactive oxygen species, caspases, p53, and NF-kB. Together, our data suggests that iNOS overexpression in tumor cells sensitizes them to low-dose L-DOX therapy, contributing to the enhanced survival of animals receiving iNOS transfection in conjunction with low doses of L-DOX.

The L-DOX dosage we opted to use (1 mg/kg) is commensurate with a human equivalent dosage that is ~25 times below the standard-of-care in high-risk NB therapy 73,74. When Cheng *et al.* dispensed three doses of thermosensitive liposomes encapsulating doxorubicin (LTLD) (0.1, 0.5, and 2.5 mg/kg) together with MR-guided high-intensity focused ultrasound (MR-HIFU) induced hyperthermia in a rabbit Vx2 tumor model, they observed that lower DOX uptake efficiencies, defined as the ratio of the accumulated tissue DOX concentration to the injected dose, correlated with higher overall doses 75. They speculated that diminishing returns occur with increasing DOX doses, possibly due to intracellular uptake saturating at higher extracellular concentrations, in agreement with earlier reports by Ranjan *et al.* 76, who used a dosage of 5 mg/kg. These data suggest that low-dosage chemotherapies can be applied with sonopermeation to augment the current standard-of-care without imparting an increased risk of systemic toxicity. Furthermore, this strategy may help reduce dosages to cut down on off-target accumulation in intermediate-risk NB where micrometastasis has not occurred. Our own findings demonstrate that higher tumoral doxorubicin concentrations and pro-apoptotic effects can be achieved at low drug doses after iNOS transfection, thus constraining the potential side effects of chemotherapy drugs. We hypothesize that this drug deposition pattern is not exclusive to doxorubicin, and can be accomplished with other chemotherapy agents and solid tumor models.

Our collective findings strongly support the argument that pre-treatment with iNOS is a clinically viable solution to improve NB response to chemotherapy. Additionally, elevated intratumoral endothelial iNOS has been linked with radiosensitivity 77, so our data holds significant implications for making tumors more amenable to radiotherapy as well as standard-of-care chemotherapies.

### Study Limitations

A limitation of this study is that the exact amount of iNOS is not quantified or controlled in the tumor. However, we have shown through semi-quantitative immunohistochemistry techniques that ultrasound gene delivery likely causes localized expression of iNOS primarily in the tumor vasculature, but also causes expression in peripheral tumor cells. Our current method is intended to observe the impacts of iNOS treatment and does not regulate it. Still, we hypothesize that more effective control can be achieved through pinpointing dosing of plasmids or by introducing on/off triggers to manage expression. Another limitation is the high dosage of pDNA and bubbles that were used in this study. To induce sufficient levels of iNOS expression for IHC analysis and to monitor tumor perfusion effects, a high amount of pDNA was systemically delivered (500 µg per mouse) as well as a high concentration of bubbles (10^9^). Non-viral gene transfection is less effective than its viral counterpart, yet we can develop methods to optimize the delivery of genetic cargo. Using larger MBs and superior cationic vectors, along with small mRNA molecules that need only penetrate the cytoplasm of endothelial cells, would likely result in an enhanced payoff for iNOS therapy.

The findings of this study suggest that repeated sonopermeation with iNOS gene therapy can reduce drug resistance in tumors, but more research is needed to fully understand the safety and efficacy of this method. In the current study, only a short time frame of 14 days was used with injections on days 0 and 7. This limits the scope of the results and does not provide a full picture of the late effects of repeated sonopermeation on tumors. Further studies should be conducted to investigate the long-term effects and safety as well as the potential for enhancing metastatic disease or other biological effects due to MB destruction. Additionally, more mice are needed to gain a better understanding of the efficacy of repeated sonopermeation. The results of this research provide a promising start, but further study is needed to determine the full potential of this method in NB treatment.

## Conclusion

The findings encompassed in this study put forth a method to overcome poor tumor perfusion and compound the efficacy of a vast array of chemotherapy, radiotherapy, and immune-based treatments. Despite our emphasis on neuroblastoma, we have concocted a versatile technology that is clinically translatable to a wide range of pediatric and adult solid tumors. In this work, we sought to pursue iNOS gene therapy to channel high levels of NO that can be produced per enzyme. In so doing we have demonstrated proof-of-principle that microbubble-mediated non-viral delivery is a rational approach to overcome major barriers associated with non-viral NO gene therapy; we expect this research to inspire the development of numerous other vascular-targeted gene therapy approaches in cancer treatment.

## Supplementary Material

Supplementary video: Projections of 3DiNOS.

## Abbreviations

αSMA: alpha smooth muscle actin
CEUS: contrast-enhanced ultrasound
CPS: contrast pulse sequencing
DOTAP: 1,2-stearoyl-3-trimethylammonium-propane
DOX: doxorubicin
EDRF: endothelium-derived relaxing factor
EPR: enhanced permeability and retention
FD: flash-destruction
FITC: fluorescein isothiocyanate
FUS: focused ultrasound
IGDD: image-guided drug delivery
IHC: immunohistochemistry
iNOS: inducible nitric oxide synthase
L-DOX: liposomal doxorubicin
MB: microbubble
NB: neuroblastoma
NO: nitric oxide
pDNA: plasmid DNA
PFB: perfluorobutane
PnP: peak-negative pressure
qCEUS: quantitative contrast -enhanced ultrasound
RBV: relative blood volume
ROI: region of interest
RR: reperfusion rate
Sono: sonopermeation
TIC: time-intensity curve
TUNEL: terminal deoxynucleotidyl transferase dUTP nick end -labeling
TV: tumor volume
UCA: ultrasound contrast agent
US: ultrasound
